# Efficacy of bougie versus balloon dilatation in children with benign esophageal stricture: a propensity score–matched retrospective cohort study

**DOI:** 10.1016/j.igie.2023.09.002

**Published:** 2023-09-17

**Authors:** Venkata Umeshreddy Devarapalli, Ujjal Poddar, Anshu Srivastava, Surender Kumar Yachha

**Affiliations:** Department of Pediatric Gastroenterology, Sanjay Gandhi Postgraduate Institute of Medical Sciences, Lucknow, Uttar Pradesh, India

## Abstract

**Background and Aims:**

Endoscopic dilatation is the primary management of esophageal strictures in children. However, pediatric data on which of the 2 endoscopic techniques, bougie or balloon, is better and the effect of endoscopic dilatation on the growth in children are lacking. We evaluated whether bougie or balloon dilatation is superior in efficacy and safety for short esophageal strictures in children and the impact of endoscopic dilatation on their growth.

**Methods:**

We performed endoscopic dilatation every 2 to 3 weeks using either a bougie or balloon and considered it adequate if we could dilate the esophageal lumen to 15 mm (11 mm in ages <2 years, 12 mm in ages 2-5 years) with complete relief of symptoms for >2 months.

**Results:**

Two hundred fifty-nine children were enrolled, with 2580 dilatations performed during the study. We performed a comparative analysis on 77 children where an exclusive bougie versus balloon dilatation was carried out for short strictures (defined as stricture length <5 cm). Both bougie and balloon dilatation showed similar efficacy (median number of sessions for adequate dilatation: 5 [interquartile range, 2.5-7.5] vs 4 [interquartile range, 2-6]; *P* = .40) and safety (perforation rate: .35% vs .54% *P* = .591) for short strictures. On a median follow-up of 17 months, there was a significant improvement in both weight and height z-scores in children with successful dilatation.

**Conclusions:**

Endoscopic bougie and balloon dilatations were safe and effective, with no significant difference for short strictures. Successful dilatation resulted in significant improvement in growth on follow-up.

Esophageal strictures are uncommon but pose a challenge for pediatric gastroenterologists worldwide. Various pediatric studies from different regions of the world have shown anastomotic stricture after esophageal atresia repair (6.7%-64.15%) and from corrosive (4.65%-49%) and peptic esophagitis (3.5%-86.6%) to be common causes in children, although these may vary geographically as well by the local referral patterns.^1^, ^2^, ^3^, ^4^ Definitive management options for esophageal strictures in children include endoscopic dilatation and surgery either by resection of a short-segment stricture or esophageal replacement.^5^^,^^6^

Endoscopic dilatation of esophageal strictures is considered the first-line therapy in children.^1^^,^^7^ In a multicenter pediatric study of 106 patients, endoscopic dilatation was shown to be safe and effective (87.73%) in the management of esophageal strictures.^4^ Evidence shows good efficacy for bougie (91%-93.7%) and balloon (79.7%-86%) dilatation in treating esophageal strictures in children; nevertheless, the lack of head-to-head comparative trials between the 2 is striking.^1^^,^^8^, ^10^ Various pediatric studies have documented that both bougie and balloon dilatations are safe, with reported perforation rates of .9% to 1.8% and 1% to 4%, respectively.^1^^,^^11^, ^12^, ^13^

Both bougie and balloon dilators have their own merits and demerits. For better application of these devices in day-to-day practice, determining which dilatation technique is superior in the management of esophageal strictures is needed, especially for short strictures in children. Also, literature is lacking on the outcome of endoscopic dilatation in relation to the etiology and nature of the stricture as well as its impact on the growth of these children.

In the present study, our primary aim was to compare the efficacy and safety of bougie versus balloon dilatation for short esophageal strictures in children. The secondary aim was to assess the impact of endoscopic dilatation on the growth of these children.

## Methods

### Patients and study design

A retrospective observational study was conducted on 259 children with esophageal strictures managed in our department between June 1994 and March 2020. All children younger than 18 years with esophageal strictures were included in the study. Clinical and endoscopic data were extracted from an electronic database and manual records, including symptoms, stricture etiology, endoscopic management, and anthropometry. We retrieved missing data as far as possible by telephone. Patients lost to follow-up before achieving adequate dilatation (unless they had an adverse event like perforation) were excluded. Then, a comparative analysis of bougie versus balloon dilatation was performed on 77 children where either an bougie (n = 54) or balloon (n = 23) dilatation was used exclusively for short strictures.

The study protocol conformed to the ethical guidelines of the 1975 Declaration of Helsinki (6th revision, 2008) as reflected in a priori approval by the Institute Ethics Committee (IEC 2021- 105-DM-EXP-38). We obtained informed consent from either parent before each procedure.

### Definitions

Adequate or successful dilatation was defined as when the esophageal lumen could be dilated to 11 mm for patients aged <2 years, 12 mm for patients aged between 2 and 5 years, and 15 mm for children aged >5 years with complete relief of symptoms for at least 2 months. Earlier studies have used 11 mm and 12.8 mm as target esophageal diameters in children younger than 5 years and have documented success.^1^^,^^11^ However, in our study, in a subgroup of children <2 years old, dysphagia subsided on dilatation to 11 mm; hence, 11 mm was taken as the endpoint for the target diameter in this age group.

Unsuccessful dilatation was defined as unresolved cases where adequate dilatation could not be achieved despite complete follow-up or where surgery was required for a difficult stricture and/or perforation. Complete follow-up was defined as successful or unsuccessful dilatation in children who remained on clinical and endoscopic follow-up.

### Treatment method

We followed a protocol-based endoscopic dilatation program in our department. Barium contrast radiography was obtained before endoscopy, and the site, number, and length of the strictures were recorded. Strictures <5 cm on a barium esophagogram were categorized as short strictures. Previous studies in children have used varying lengths of <2 cm and <5 cm for defining short strictures; we chose the latter based on larger pediatric studies that showed long strictures (>5 cm) had poor outcomes and increased risks of adverse events.^1^^,^^5^^,^^7^^,^^14^

Dilatation was performed at 2- to 3-weekly intervals using either polyvinyl bougies or controlled radial expansion fixed and wire-guided balloon dilators. We followed our department's dilatation protocol during our entire study duration. All dilatations were performed with patients under conscious sedation using intravenous ketamine (1-2 mg/kg) and midazolam (.1 mg/kg). Children were monitored during and for 4 to 6 hours after -dilatation.

For bougie dilatation, American Bard dilators (Bard Medical, Franklin Lakes, NJ, USA) were used starting from 5 mm up to 15 mm, with increments of 1-mm diameter. After an endoscopically placed guidewire, bougie dilators were passed under fluoroscopic guidance, and during each session, serial dilators were used depending on the tightness of the stricture using the rule of 3 (ie, in a single session, ≤3 bougie dilators of sequentially larger size were passed once moderate or greater resistance was evident).

For balloon dilatation, controlled radial expansion fixed (with a balloon length of 8 cm) or wire-guided balloon (length of 5.5 cm) dilators (Boston Scientific, Marlborough, Mass, USA) were used with an initial size beginning from 6 mm up to 15 mm. Balloon dilatation was performed using an endoscopically placed fixed or wire-guided balloon dilator kept inflated for 2 to 3 minutes with a check on waist disappearance under fluoroscopy. We inflated the balloon by increasing the balloon inflation pressure as per the manufacturer’s protocol. We monitored the expansion of the balloon by direct endoscopic vision and fluoroscopically by inflating with a radiopaque contrast targeting waist disappearance using the maximal intended diameter for the session. For tight strictures (when a 5-mm bougie could not be negotiated), a Soehendra biliary dilatation catheter (Cook Medical, Bloomington, Ind, USA) was used for the initial negotiation, followed by bougie dilatation.

Endoscopist preference and length of the stricture were factors in deciding the choice of the dilator. The overall endoscopic experience of using bougie and balloon dilators differed for different endoscopists. However, for a given endoscopist, when using a bougie or a balloon dilator, all had the same experience using both tools. We used the same equipment throughout the study duration for both bougie and balloon dilatation.

Once adequate dilatation was achieved, subsequently, dilatations were performed on an "as-needed" basis. For a more uniform assessment of the frequency of dilatation, the periodic dilatation index (PDI) was calculated as the number of dilatations required divided by the duration of time in months.^15^ The PDI was calculated from the first dilatation to the adequate dilatation and from that point until the last follow-up, in units of dilatations per month.

### Comparison of bougie versus balloon dilatation

We compared both dilator types in a single, short esophageal stricture for uniformity, because a long stricture is preferentially dilated by a bougie. Children with successful dilation who underwent either an exclusive bougie or balloon dilatation for short strictures were compared for efficacy and safety. Efficacy was assessed by the number of sessions needed for adequate dilatation and safety by the perforation rate.

### Assessment of growth

We evaluated growth in children where baseline and follow-up data for weight and height were available. Baseline data constituted weight and height at the first dilatation, whereas follow-up data were when the target dilatation was achieved. We calculated baseline and follow-up weight and height z-scores using World Health Organization Anthro and Anthroplus software (WHO, Geneva, Switzerland). We used the z-score for comparison rather than absolute values of weight and height because it is the standard of care for assessing the nutritional status in children and forms a common variable that is representative of all children with varying ages in the cohort. Changes (follow-up minus baseline) in weight and height z-scores were used for comparing the effects of balloon versus bougie dilatation on growth. Also, the proportion of children with successful dilatation who were less than –2 standard deviations (SDs) for z-score for weight and height at baseline and follow-up was evaluated overall and in the etiologic subgroups.

### Comparison by etiology

Anastomotic and corrosive strictures were compared to assess the stricture responsiveness for both etiologies. The number of sessions required for adequate dilatation, PDI, and the requirement and number of as-needed sessions for anastomotic and corrosive strictures were assessed. In children with anastomotic and peptic strictures, a proton pump inhibitor was prescribed until stricture resolution. Regular assessment for any features suggestive of reflux disease requiring the ongoing need for acid suppression therapy was made.

### Comparison by nature of the stricture

A comparison of short strictures (<5 cm in length) versus long and/or multiple strictures was performed only for corrosive strictures, because the other etiologies commonly present with a short stricture.

### Statistical analysis

Data were analyzed using SPSS statistical package version 23 (SPSS Inc, Chicago, Ill, USA) and R Statistical Package 3.6 (R Foundation, Indianapolis, Ind, USA). Continuous data are expressed as median and interquartile range (IQR). Categorical variables were compared by using the χ^2^ test or Fisher exact test and continuous variables by using the Mann-Whitney test. We performed propensity matching (for age, with .05 as the tolerance limit) with multivariate analysis to compare exclusive bougie versus balloon dilatation groups. A comparison of baseline and follow-up growth was performed using the Kruskal-Wallis H test. *P* < .05 was taken as significant.

## Results

The median age of 259 children with symptomatic esophageal strictures was 2.5 years (IQR, 1.1-5.5), with a male-to-female ratio of 2.2:1. Among them, 188 had a single, short esophageal stricture in which an exclusive bougie or balloon dilatation was performed in 77.

**Figure 1:**
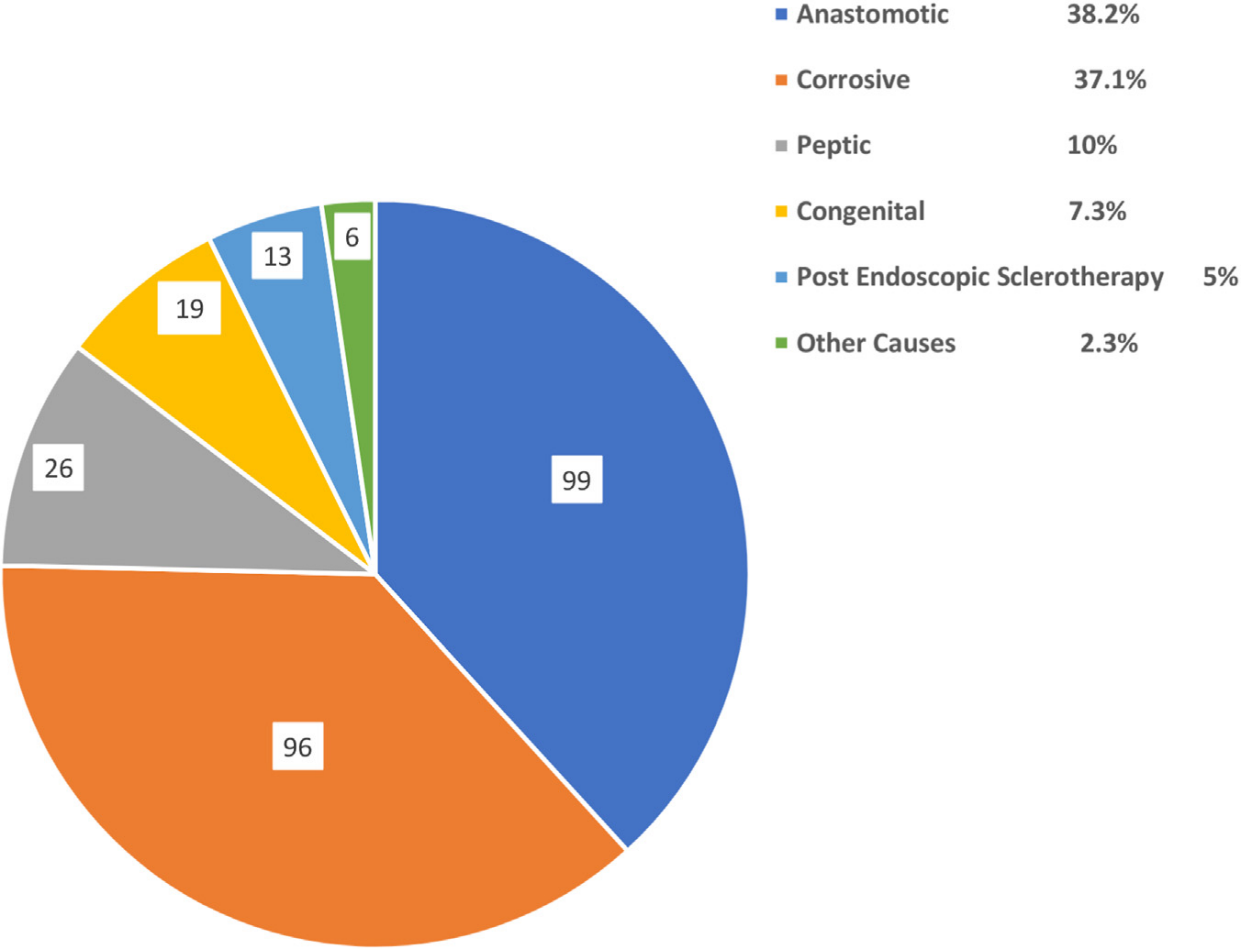
Pie chart showing the etiologic spectrum of the overall cohort.

As expected, all children presented with dysphagia. Data were missing in some patients regarding different symptoms. Vomiting was seen in 163 of 207 patients (78.74%), respiratory issues in 48 of 199 (24.1%), and failure to thrive in 109 of 196 (57.40%). Among the etiology of strictures, the most common was anastomotic (99 [38.2%]), closely followed by corrosive (96 [37.1%]), peptic (26 [10%]), congenital (19 [7.3%]), postendoscopic sclerotherapy (13 [5%]), and other (6 [2.3%]) (Fig. 1). Among the anastomotic strictures, 96 were after esophageal atresia repair and 3 were after surgery for esophageal perforation (corrosive, 2; unknown, 1). In the corrosive group, acid ingestion was responsible for 49 strictures, alkali for 34, and the remaining 13 were unknown. Among the other causes, 2 strictures were from Steven Johnson syndrome, 1 from epidermolysis bullosa, 1 after boiled water ingestion, 1 from suspected connective tissue disorder, and 1 of unknown etiology.

### Efficacy of endoscopic dilatation

**Figure 2:**
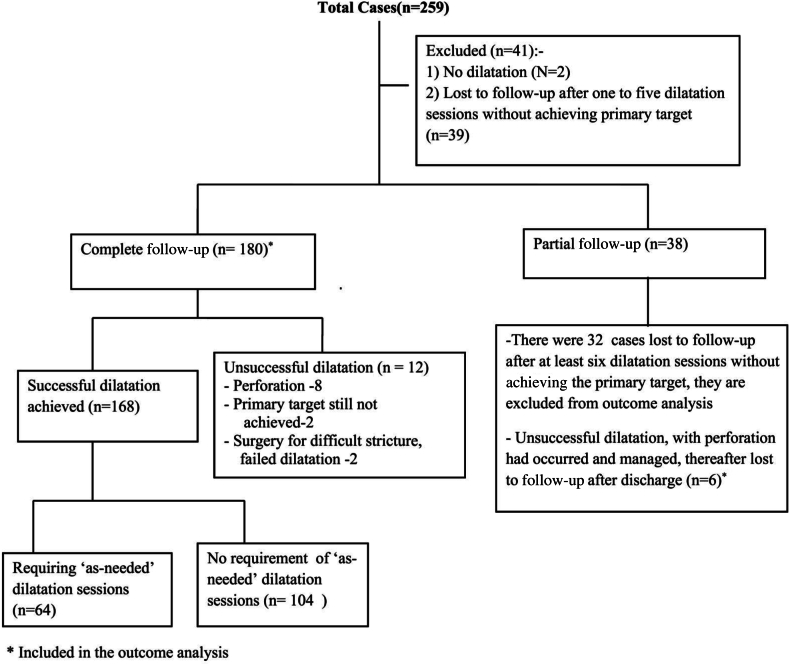
Flowchart showing outcomes of the overall cohort.

Of the 259 cases, 41 were lost to follow-up after ≤5 dilatation sessions and 32 after ≥6 sessions but before achieving adequate dilatation (Fig. 2). The outcome was analyzed for 186 cases; dilatation was successful in 168, with an efficacy of 90.32%. Of the 18 unsuccessful cases, perforations occurred in 14 children, 2 children with unresolved strictures were continuing on dilatation at the time of the last follow-up, 1 child underwent a 2-staged colonic interposition, and another child was scheduled for esophageal replacement surgery for difficult stricture with failed dilatation.

Among the successfully dilated cases, we did a comparative analysis in 77 patients with short stricture where we could perform either an exclusive bougie dilatation (n = 54) or exclusive balloon dilatation (n = 23). On univariate analysis, the number of dilatation sessions required for adequate dilatation was significantly better for the balloon group than the bougie group; however, age was found to be a significant confounding factor, with younger children more often dilated with a bougie. Hence, propensity matching for age was performed. Multivariate analysis after propensity matching (balloon, 23; bougie, 20) showed no significant difference in the median number of sessions required for adequate dilatation in both groups (Table 1). Similarly, no significant differences were found concerning the requirement of as-needed dilatation sessions, the median number of as-needed sessions, and the median PDI (dilatations per month) after adequate dilatation.

**Table 1:** Comparison between balloon and bougie dilatation for short strictures

Parameter	Balloon (n = 23)	Bougie (n = 54)	*P* value
Age, y	3 [1.08-5] (.16-15)	1.25 [.48-2.5] (.08-13)	.017∗
Male sex	15 (65.22)	35 (64.81)	.869
Weight z-score at presentation	–2.02 [–3.10 to –.71] (–4.63 to –.17)	-2.41 [–3.80 to –1.74] (-4.55 to –.14)	.251
No. of sessions for adequate dilatation	4 [2-6] (1-18)	6 [3-9] (1-46)	.035∗
PDI before adequate dilatation	1 [.50-1.45] (.33-2)	1.4 [.98-1.70] (.18-2.57)	.021∗
Requirement for as-needed sessions (yes)	3 (13.04)	18 (33.33)	.069
No. of as-needed sessions	1 [1-2.75] (1-2)	2 [1-5] (1-19)	.070
PDI after adequate dilatation	.5 [.50-.50] (.50-1.00)	.5 [.27–.64] (.10-1.00)	.485
Perforation rate for short strictures (balloon, 23; bougie, 54)	3/560 (.54%)	3/856 (.35%)	.591
Perforation rate (for the overall cohort, n = 259)	5/970 (.51)	6/1610 (.37)	.595
Change in weight z-score (follow-up minus baseline weight z-scores)	.61 [.35-1.12] (–.82 to 3.97)	1.57 [.17-2.17] (–1.11 to 4.00)	.459
Change in height z-score (follow-up minus baseline height z-scores)	.64 [–.01 to 1.39] (–1.97 to 3.25)	.80 [.19-2.01] (–2.52 to 4.35)	.604
*Multivariate analysis (after propensity matching)*			
**Parameter**	**Balloon (n = 23)**	**Bougie (n = 20)**	***P* value**
No. of sessions for adequate dilatation	4 [2-6] (1-18)	5 [2.5-7.5] (1-46)	.40
PDI before adequate dilatation	1 [.50-1.45] (.33-2)	1 [.50-1.45] (.18-2.50)	.710

Values are median [interquartile range] (range) or n or n/N (%).

*PDI*, Periodic dilatation index.

∗ *P* value is significant (*P* < .05).

### Safety of endoscopic dilatation

In the overall cohort of 259 patients, desaturation events occurred in 11 of 2580 sessions; 8 (out of 970 sessions) were during balloon dilatation and 1 each was sedation-related, during bougie dilatation (out of 1610 sessions), and during insertion of an adult (8.6-mm) gastroscope (GIF Q180, Olympus Optical Co, Tokyo, Japan). Desaturation was transient in all cases, with spontaneous improvement in 8 patients and 3 requiring brief bag and mask ventilation. All cases were successfully continued on the dilatation program.

The risk of perforation was assessed by the perforation rate, calculated as the total number of perforations divided by the total number of dilatation sessions. In the overall cohort of 259 patients, 14 esophageal perforations (guidewire-related, 3; bougie, 6; balloon, 5) occurred during 2580 dilatation sessions (.54%). The perforation rate in the overall cohort (n = 259) for balloon dilatation was .51% (5/970) and that for bougie dilatation was .37% (6/1610), which was not significant (*P* = .595). Of the 14 perforations, 2 were managed conservatively and 12 were managed surgically; 6 patients were dysphagia free on follow-up, 1 required subsequent dilatation for anastomotic stricture, 1 child died postoperatively because of sepsis, and the remaining 4 children were lost to follow-up after surgery.

In the subgroup of 77 patients where an exclusive bougie or balloon dilatation was performed for short strictures, 7 esophageal perforations (guidewire-related, 1; bougie, 3; balloon, 3) occurred over 1416 dilatation sessions. There was no significant difference (*P* = .591) in the perforation rate for balloon (3/560 [.54%]) and bougie (3/856 [.35%]) dilatation for short strictures.

### Impact of endoscopic management on growth

**Figure 3:**
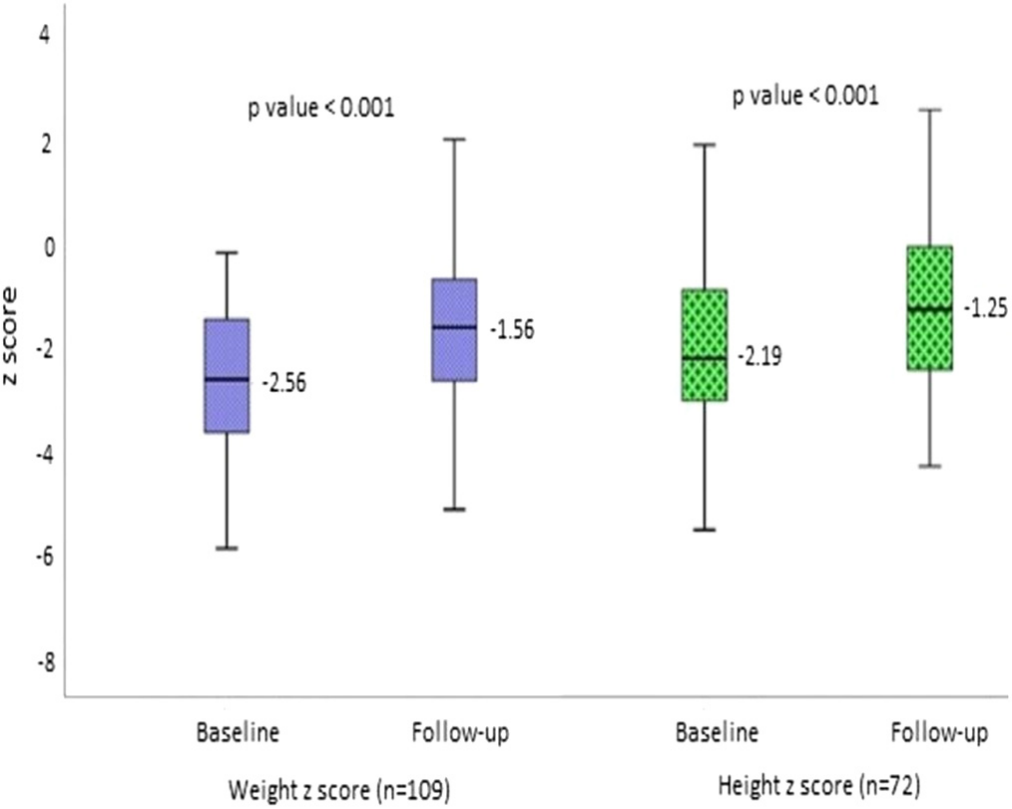
Box plots comparing baseline and follow-up weight and height z-scores in children with successful dilatation.

Over a median follow-up of 17 months (IQR, 6-39), paired data (baseline and follow-up [when target dilatation had been achieved]) for weight and height were available for 109 and 72 children, respectively. Those with successful dilatation showed a significant improvement in their weight and height z-scores from baseline to follow-up overall (Fig. 3) as well as across the 3 etiologic groups (Supplementary Table 1, available online at www.igiejournal.org). Also, for successful dilatation, overall, a significant improvement was found in the proportion of children who were less than a –2 SD z-score for weight (62% vs 39%, *P* < .001) and height (53% vs 33%, *P* = .019) from baseline to follow-up.

In the proportion of children showing improvement who were less than a –2 SD z-score for weight and height by etiologic subgroups (anastomotic, corrosive, and remaining strictures), significant improvement was documented in children in the anastomotic stricture group for weight z-score of –2 SD (65% vs 40%, *P* = .015) and height z-score of –2 SD (48% vs 18%, *P* = .009). However, the change in proportion for both weight and height z-scores was not significant in the corrosive stricture group (weight z-score –2 SD [53% vs 34%, *P* = .108], height z-score –2 SD (57% vs 48%, *P* = .542]) and the remaining stricture group (weight z-score –2 SD [74% vs 48%, *P* = .073] and height z-score –2 SD [56% vs 44%, *P* = .511]). In the subgroup of 77 children in which an exclusive bougie (n = 54) or balloon (n = 23) dilator was used, there was no significant difference in the change in weight (median, .61 vs 1.57, respectively; *P* = .459) and height (median, .64 vs .80, *P* = .604) z-scores between the balloon and bougie groups.

### Comparison of anastomotic versus corrosive stricture

The comparisons between anastomotic and corrosive strictures are shown in Table 2. Compared with the corrosive group (n = 55), children with anastomotic strictures (n = 76) presented at a younger age (*P* < .001) and required significantly fewer sessions to achieve adequate dilatation (*P* < .001). Also, the requirement for (*P* = .003) and number of as-needed sessions (*P* = .018) were significantly less in the anastomotic group. Comparison of anastomotic (n = 42) and corrosive (n = 18) strictures in the bougie alone group versus balloon alone group showed a younger age at presentation in the anastomotic group (median, .83 years vs 2.75 years, respectively; *P* < .001). There was no significant difference in the median number of sessions (5 vs 7, *P* = .23) before achieving adequate dilatation. The median number of as-needed sessions (1 vs 3, *P* = .04) was significantly less in the anastomotic group. However, there was no significant difference in the requirement of as-needed sessions (19.05% vs 38.89%, *P* = .11) and median PDI (dilatations per month) after adequate dilatation (.5 vs .5, *P* = .30) among the 2 groups.

**Table 2:** Comparison between anastomotic and corrosive strictures

Variable	Anastomotic stricture (n = 76)	Corrosive stricture (n = 55)	*P* value
Age, y	.87 [.25-1.96] (.08- 15)	3 [2-4] (.58-17)	<.001∗
Male sex	48 (63.16)	37 (67.27)	.71
No. of sessions for adequate dilatation	7 [4-10] (1-34)	11 [7-19] (2-83)	<.001∗
PDI before adequate dilatation	1.36 [.94-1.70] (.33-2.5)	1.28 [.90-1.67] (.18-3)	.83
Requirement for as-needed sessions (yes)	20 (26.31)	29 (52.73)	.003∗
No. of as-needed sessions	1 [1-3.5] (1-33)	4 [1.75-7.25] (1-20)	.018∗
PDI after adequate dilatation	.5 [.5-.73] (.22-2)	.5 [.24-.83] (.1-1.5)	.112

Values are median [interquartile range] (range) or n (%).

*PDI*, Periodic dilatation index.

∗ *P* value is significant (*P* < .05).

### Comparison of short versus long and/or multiple strictures

Among the corrosive stricture group, a comparison of short versus long and/or multiple strictures showed a younger patient age at presentation among those with short strictures (median, 3 years [IQR, 2-3] vs 3.5 years [IQR, 2.50-6]; *P* = .04] (Supplementary Table 2, available online at www.igiejournal.org). The median number of sessions to achieve adequate dilatation was significantly less in the group with a short stricture (9 [IQR, 5-13] vs 19 [IQR, 12.25-28.50], *P* < .001). An increased requirement was found for as-needed sessions in the group with long and/or multiple strictures (9/31 [29.03%] vs 20/24 [83.33%], *P* < .001).

Feeding gastrostomy or jejunostomy was used for supplementing feeds in 3 of 168 children (all corrosive strictures) who underwent successful dilatation. A Soehendra biliary dilatation catheter was used in 36 cases (corrosive, 17; anastomotic, 13; congenital esophageal stenosis, 4; peptic, 1; other, 1) for the initial negotiation of a tight stricture.

## Discussion

In the present study, we compared bougie versus balloon dilatation for short esophageal strictures in children and assessed the impact of endoscopic dilatation on their growth. Our results showed that both bougie and balloon dilatation had similar efficacy and safety for short strictures. In successfully dilated children, we documented a significant improvement in their growth on follow-up.

Our results have shown that overall anastomotic (38.2%) and corrosive (37.1%) strictures were the 2 most common etiologies, similar to other reported studies worldwide.^2^^,^^4^^,^^5^^,^^8^ In the overall cohort of 259 patients, we documented that endoscopic management by bougie and balloon dilatation was safe and effective for esophageal strictures, with 90% achieving successful dilatation. Although similar results were reported in previous studies using bougie (91%-93.7%) and balloon (79.7%-86%) dilatation for the management of esophageal strictures in children, our study adds to the existing literature as one of the largest in terms of cohort size.^1^^,^^8^, ^10^ Also, we documented success over a longer study duration and in a diverse etiology of esophageal strictures. The overall rate of perforation (.54%; bougie, .37%; balloon, .51%) in our study is almost similar to or less than the reported figures of .9% to 1.8% for bougie dilatation and 1% to 4% for balloon dilatation.^1^^,^^11^, ^12^, ^13^

Studies comparing bougie and balloon dilatation for esophageal strictures are scarce, especially in children. In a pediatric study comparing bougie and balloon dilatation at 2 different periods retrospectively, balloon dilatation (n = 16) was significantly better than bougie dilatation (n = 12), requiring fewer dilatation sessions in anastomotic strictures.^16^ We compared 77 children with exclusive bougie or balloon dilatation performed for short strictures. We demonstrated using propensity-matched multivariate analysis that both dilators have comparable efficacy and safety for short strictures in children. With no clear superiority between balloon and bougie dilators, endoscopists may choose either dilator type for short strictures in children until further evidence, especially from randomized trials, is obtained. For uniformity, we compared both dilator types for only single, short esophageal strictures (length <5 cm). In long strictures, using a balloon dilator would require dilatation sequentially at multiple levels of stricture; hence, it is uncommon that long strictures are exclusively managed by balloon dilatation. A bougie dilator exerts an axial force on the stricture, theoretically posing a greater risk of mucosal injury and perforation. On the other hand, a balloon dilator has the property of exerting a radial force at the stricture site. Other advantages of the balloon dilator are greater precision because it dilates only the exact narrowing and the possibility of visualizing the dilatation process endoscopically as it occurs.^3^^,^^5^

Chang et al,^14^ in a study of 50 children with most commonly anastomotic strictures (42%) followed by corrosive strictures (26%), found significant improvement in the weight for age z-scores after endoscopic balloon dilatation, particularly in those with short strictures in the middle esophagus. However, no study compared the growth parameters by specific etiology, which could help targeted nutritional rehabilitation. Our study demonstrated that over a median follow-up of 17 months, children with successful dilatation had significant improvement in their weight and height z-scores for all etiologies, suggesting our age-related target dilatation was adequate for catch-up growth. Comparing the proportion of children with successful dilatation who were less than a –2 SD z-score for weight and height at baseline to follow-up, there was a significant improvement overall and in the anastomotic subgroup. For anastomotic strictures, this is likely a direct result of the fewer initial and as-needed sessions required in achieving the target diameter, allowing the child to have a regular diet with required calories early. The improvement was not significant in the corrosive stricture group, mainly because these strictures are rigid and often refractory, requiring multiple sessions to achieve and maintain a target lumen diameter. Also, these children may have an associated pyloric stricture and possible psychiatric issues that may affect their growth. Other factors that could affect the outcome include the prompt use of proton pump inhibitors where indicated, nutritional rehabilitation, and maintaining a regular follow-up.^17^^,^^18^

The number of sessions required for adequate dilatation and the requirement and median number of as-needed sessions were significantly less for anastomotic than for corrosive strictures in our study. Anastomotic strictures are short and pliable, unlike corrosive injuries, which cause deep burns and dense cicatrization of the esophageal wall, giving rise to strictures that are rigid and difficult to dilate.^19^^,^^20^ Previous studies have reported that long corrosive strictures (>5 cm) required a significantly higher number of sessions for adequate dilatation than short strictures.^1^^,^^11^ Long strictures are complex, usually tortuous, and challenging to dilate.^21^^,^^22^ We demonstrated that dilatation was successful in long and/or multiple strictures, albeit with a higher number of sessions for adequate dilatation and an increased requirement for as-needed sessions. In tight strictures, not allowing the smallest size bougie or balloon dilator to pass, a Soehendra biliary dilatation catheter is a useful accessory, particularly for the initial sessions in negotiating a tight stricture.^23^

The limitations of our study are that of any retrospective study, which precludes a completely unbiased comparison. The choice of dilators depended on the endoscopist's choice and was not randomized. Also, the different experiences among endoscopists in using both tools could have affected the outcome. Although statistical analysis with propensity matching was used to adjust the selection and confounding biases, the resulting sample size was small, which could have underpowered the outcome. Future studies should be randomized clinical trials with a larger sample size for comparing bougie and balloon dilatation. Also, despite the fact that the treatment methods were standardized, it is possible that the long enrollment period of the study participants could have affected the treatment responses at different time points. A significant proportion of cases were lost to follow-up before achieving the target, which might have influenced the outcome data. However, our accurate electronic record-keeping and good follow-up allowed us to study the interventions in detail.

In conclusion, for esophageal strictures, both endoscopic balloon and bougie dilatations were safe and effective, with no significant difference for short strictures in children. Successful dilatation resulted in significant improvement in growth on follow-up.

## Disclosure

All authors disclosed no financial relationships.

## Figures and Tables

**Supplementary Table 1:** Comparison of baseline and follow-up weight and height z-scores in children with successful dilatation

	Parameter	Baseline	Follow-up	*P* value
Overall (n = 1 6 8) (paired data weight z-scores, n = 109; height z-scores, n = 72)	Weight z-score	–2.56 [–3.58 to –1.40] (–.13 to -9.36)	–1.56 [–2.6 to –.64] (–5.06 to 2.05)	<.001∗
	Height z- score	–2.19 [–3.0 to –.87] (–5.47 to 1.91)	–1.25 [–2.42 to –.17] (–4.25 to 2.58)	<.001∗
Anastomotic stricture (n = 76) (paired data weight z-scores, n = 48); height z-scores, n = 33)	Weight z-score	–3.01 [–3.82 to –1.26] (–4.63 to –.13)	–1.44 [–2.67 to –.31] (–4.02 to 2.05)	<.001∗
	Height z- score	–1.81 [–3.07 to –.87] (–4.07 to 1.63)	–1.09 [–1.74 to –.13] (–3.55 to 2.02)	.001∗
Corrosive strictures (n = 55) (paired data weight z-scores, n = 38; height z-scores, n = 21)	Weight z-score	–2.05 [–3.33 to –1.29] (–9.36 to –.14)	–1.55 [–2.43 to –.62] (–5.06 to –.75)	<.001∗
	Height z- score	–2.26 [–3.05 to –.71] (–5.20 to 1.91)	–1.93 [–2.92 to –.06] (–4.17 to 2.58)	.045∗
Remaining strictures (n = 37) (paired data weight z-scores, n = 23; height z-scores, n = 18)	Weight z-score	–2.73 [–4.05 to –1.89] (–5.81 to –.17)	–1.76 [–3.03 to –.90] (–4.65 to 1.47)	<.001∗
	Height z- score	–2.23 [–3.16 to –.96] (–5.47 to .90)	–1.87 [–2.59 to –.53] (–4.25 to 1.05)	.044∗

Values are median [interquartile range] (range).

∗ *P* value is significant (*P* < .05).

**Supplementary Table 2:** Corrosive strictures: comparison between short and long and/or multiple strictures

Variable	Short (n = 31)	Long/multiple (n = 24; long = 24; multiple = 15)	*P* value
Age, y	3 [2-3] (.58-17)	3.5 [2.50-6] (1, 2, 3, 4, 5, 6, 7, 8, 9, 10, 11, 12, 13, 14, 15, 16, 17)	.040∗
Male sex	19 (61.29)	18 (75)	.387
No. of sessions for adequate dilatation	9 [5-13] (2-46)	19 [12.25-28.50] (6-83)	<.001∗
PDI before adequate dilatation	1.33 [1-1.80] (.18-2.57)	1.21 [.88-1.60] (.46-3)	.481
Requirement for as-needed sessions (yes)	9 (29.03)	20 (83.33)	<.001∗
No. of as-needed sessions	3.5 [1.75-7.25] (1-10)	4.5 [1.25-9.25] (1-20)	.639
PDI after adequate dilatation	.5 [.22-.88] (.10-1.25)	.5 [.25-.82] (.10-1.50)	.904

Values are median [interquartile range] (range) or n (%).

*PDI*, Periodic dilatation index.

∗ *P* value is significant (*P* < .05).
